# Hospitalized patients and stimulant use-associated heart failure: importance of ejection fraction and related risk factors

**DOI:** 10.3389/fcvm.2025.1566481

**Published:** 2025-07-03

**Authors:** Akshat Agrawal, Brooke Scardino, Diensn G. Xing, Md. Shenuarin Bhuiyan, Rick A. Bevins, Kalgi Modi, Tarek Helmy, Steven A. Conrad, Nicholas E. Goeders, Md Mostafizur Rahman Bhuiyan, John A. Vanchiere, A. Wayne Orr, Christopher G. Kevil, Mohammad Alfrad Nobel Bhuiyan

**Affiliations:** ^1^ Department of Public Health, Louisiana State University Health Sciences Center at Shreveport, Shreveport, LA, United States; ^2^ Department of Medicine, Louisiana State University Health Sciences Center at Shreveport, Shreveport, LA, United States; ^3^ Department of Molecular and Cellular Physiology, Louisiana State University Health Sciences Center at Shreveport, Shreveport, LA, United States; ^4^ Department of Pathology and Translational Pathobiology, Louisiana State University Health Sciences Center at Shreveport, Shreveport, LA, United States; ^5^ Department of Psychology, University of Nebraska-Lincoln, Lincoln, NE, United States; ^6^ Department of Pediatrics, LSU Health Sciences Center at Shreveport, Shreveport, LA, United States; ^7^ Department of Pharmacology, Toxicology & Neuroscience, Louisiana State University Health Sciences Center at Shreveport, Shreveport, LA, United States; ^8^ Department of Pediatric Cardiology, Bangabandhu Sheikh Mujib Medical University, Dhaka, Bangladesh; ^9^ Louisiana Addiction Research Center, Louisiana State University Health Sciences Center at Shreveport, Shreveport, LA, United States

**Keywords:** stimulant, HFpEF, HFrEF, trend, disparity

## Abstract

**Background:**

Methamphetamine and cocaine use are known risk factors for heart failure (HF). Previous studies focused on HF cases identified as either methamphetamine or cocaine-induced HF with no study identifying the HF subtype most associated with stimulant use. Our study hypothesizes that stimulant users have a higher odds of developing HFrEF than HFpEF. Our study also compares demographic and comorbidities between the HF subtypes.

**Methods:**

National Inpatient Sample data from 2008 to 2020 were used to identify hospital admissions among stimulant users with HF. The chi-square test for categorical variables and *t*-test for continuous variables was used for the weighted sample. *P*-value was found by linear trend analysis. The trend stratified by age, sex, race, and United States region (defined by the US Census Bureau) was analyzed by the Cochran-Armitage trend test. A generalized linear model determined the HF subtype related to stimulant use adjusted for traditional risk factors, and another model estimated vulnerable patient characteristics.

**Results:**

Stimulant use was more likely to be associated with HFrEF (OR = 1.97, CI 1.93–2.01), while less associated with HFpEF (OR = 0.96, CI 0.94–0.98). HF among stimulant users was common (*p* *<* 0.001) in males, those aged 41–64, Black patients, Medicaid users, those in the <50 percentile income, and the South or West regions. Stimulant-related HF hospitalizations increased significantly from 2008 to 2020 for all subcategories (*p* *<* 0.001).

**Conclusion:**

Stimulant use is positively associated with HFrEF, with the highest risk being in those middle-aged, male, Black, or covered by Medicaid. The higher likelihood of traditional risk factors for HF in stimulant-related HF supports the hypothesis that stimulants induce multifactorial damage to the cardiovascular system.

## Introduction

Cocaine and methamphetamine are highly addictive substances causing increasing morbidity and mortality in the US. Methamphetamine-related hospital admissions have increased by over 6.73-fold in the past 30 years, along with increasing mortality, highlighting the growing public health problem of stimulant use and misuse (1–3). Unfortunately, in contrast to opioid overdose, stimulant (methamphetamine and cocaine) overdose has no direct pharmacological treatment (4). To advance our understanding of the causes and consequences of stimulant use as we search for better treatment outcomes, it is critical to characterize the trends and pathophysiology of stimulant misuse and associated overdoses. Heart failure (HF) is a critical outcome of chronic stimulant use, with methamphetamine and cocaine contributing to cardiotoxicity.

Studies have suggested methamphetamine and cocaine cause myocardial injury through chronic catecholamine excess (5–8). Methamphetamine causes direct myocardial toxicity through oxidative stress and increased apoptosis, contributing to chronic inflammation leading to loss of myocytes, cardiomyocyte hypertrophy, and myocardial fibrosis, thus impairing systolic function (7, 9–16). Cocaine similarly causes myocyte injury via catecholamine excess with associated ventricular hypertrophy and reduced ventricular compliance, which causes inadequate ventricular filling and diastolic failure (17). Cocaine also causes cardiomyopathy by inhibiting sodium channels in the myocardial tissue, decreasing contractility (18).

HF can be classified by ejection fraction: reduced ejection fraction (HFrEF) or preserved ejection fraction (HFpEF). Methamphetamine and cocaine have been traditionally thought to cause HFrEF, with recent work highlighting its association with HFpEF as well (17, 19, 20). Despite this, there is surprisingly limited understanding of the clinical burden posed by different stimulant-associated HF subtypes. Furthermore, stimulant-related outcomes and heart failure outcomes vary widely between different demographic groups, with minority groups having a disproportionate disease burden and worse outcomes in both ejection fraction conditions (21–24).

Across studies, most cases were reported as methamphetamine-induced HF, cocaine-induced HF, or stimulant-induced HF, with only a few studies distinguishing between HFrEF and HFpEF. The outcome of this latter work was mixed (18–20, 25, 26). In our extensive literature search, there was no study that estimates which subtype of HF is more likely to be associated with stimulant use. Accordingly, the present study will provide an estimate of this association between stimulant use and HF subtype. Based on findings from previous studies, we hypothesize that HFrEF will be more likely to be associated with stimulant use than HFpEF. Additionally, our study will compare patient characteristics and comorbidities among stimulant-using HF hospitalizations by subtype. We have combined methamphetamine, amphetamine, and cocaine users and defined them as stimulant users in our study, given their common pathophysiology.

## Methods

Hospital admissions among methamphetamine, amphetamine, and cocaine users with HF from 2008 to 2020 were quantified using data from the National Inpatient Sample (NIS), Healthcare Cost and Utilization Project (HCUP), and Agency for Healthcare Research and Quality (27). The NIS is the largest publicly available database for inpatient care, encompassing patient information from 7 million annual hospital stays in the United States, representing about 97% of Americans. The NIS currently uses the total number of discharges in each stratum to determine discharge weights instead of using the number of hospitals to get national discharge weights, thus reducing the error for national estimates by 42–48 percent. This current discharge method was retroactively applied to the NIS data before 2012 to adjust discharge weights, allowing for trend exploration using these techniques (27). Each sample in the NIS database represents a single patient hospitalization and over 100 clinical elements such as patient diagnoses, demographics, and aspects of hospital stay. In this study, we isolated adults (>18 years) who were hospitalized with a primary or secondary diagnosis of heart failure and stimulant use (methamphetamine, amphetamine, or cocaine) with the International Classification of Diseases Coding System (ICD) 9 and 10. Further explanation of ICD codes for HF and stimulant use is shown in Supplementary Tables S1, S2. The NIS data are deidentified, and the Institutional Review Board (IRB) at LSU Health Sciences Center, Shreveport, determined that this study was exempt from IRB oversight.

### Statistical analysis

National estimates were derived using survey-specific statements and patient-level weights. Prior to 2012, trend weight was used for national estimates of trend analysis; however, following 2012, regular discharge weight methods were used to find national estimates. All discharge weights are the same for an NIS year's hospital discharges. Therefore, trend weight files were merged with initial NIS files by year and hospital ID. Frequencies and percentages of categorical variables were analyzed for unweighted (raw hospital admission data) and weighted (nationally representative data) hospital admissions. NIS sample design and weight were examined using design-based Chi-Square tests for categorical values and designed-based t-tests for continuous data (28). Missing values did not surpass 5%, so imputation was not required. A generalized linear model found the 95% confidence interval and odds ratio. The first model was designed to estimate the likelihood of being hospitalized with either HFrEF or HFpEF given stimulant use. The model was adjusted for gender, age, race, primary payer, median household income, length of hospital stays, region in the US, ischemic heart disease, diabetes, hypertension, obesity, alcohol use, renal failure, and year of data collected (29). Using modifications like the first model, the second model was designed to determine at-risk demographic groups and associated risk factors among stimulant-using HFpEF and HFrEF patients. The *p*-value for trends was found by linear trend analysis. The trend stratified by age, sex, race/ethnicity, and hospital region was then analyzed using the Cochran-Armitage trend test. The states included in each hospital region is available in Supplementary Table S3. Design and analytical guidelines have already been described for NIS data, and HCUP standards were followed for analysis (30–33). All statistical tests were calculated using open-source software R (version 4.4.1). Statistical significance was set at *p* < 0.05.

## Results

### Demographic, risk factors, and trend analysis results for HFpEF hospital admissions

HFpEF hospitalizations among stimulant users [HFpEF(+Stim)] increased by 423% from 2008 to 2020 (*p* *<* 0.001). HFpEF hospitalizations in stimulant non-users [HFpEF(-Stim)] were significantly (*p* *<* 0.001) more prevalent in female patients (60.79%), and HFpEF(+Stim) were more prevalent in male patients (59.5%). Trend analysis showed HFpEF(+Stim) significantly (*p* *<* 0.001) increased from 2008 to 2020 in males (409%) and females (446%), with males comprising the majority (*p* *<* 0.001).

Hospitalizations also differed significantly (*p* *<* 0.001) by age. HFpEF(-Stim) was most increased in patients aged >64 years (78.17%), whereas HFpEF (+Stim) was highest in those aged 41–64 years (76.52%) with hospitalizations increasing significantly (*p* *<* 0.01) from 2008 to 2020.

By race/ethnicity, HFpEF(-Stim) was significantly higher (<0.001) in non-Hispanic White (hereafter White) patients (73.36%). In contrast, HFpEF(+Stim) was comprised of primarily non-Hispanic Black (hereafter Black) patients (53.46%), followed by White patients (34.07%). Trend analysis revealed that HFpEF(+Stim) had a significantly (*p* *<* 0.001) higher burden among White and Black patients, increasing significantly (*p* *<* 0.001) from 2008 to 2020.

Regarding healthcare coverage, Medicare constituted 80.71% of HFpEF(-Stim) admissions. HFpEF(+Stim) was more evenly distributed between Medicare (35.49%) and Medicaid (44.68%). Further, HFpEF(+Stim) were more likely to self-pay (7.79%) than non-users (1.43%). Household income was also associated with stimulant use and HF (*p* *<* 0.001). In HFpEF(-Stim), there was a relatively uniform distribution between different percentile household incomes. In contrast, HFpEF(+Stim) individuals mostly belonged to the lowest quartile of household income (52.71%). Both HFpEF(+Stim) (36.32%) and HFpEF(-Stim) (37.3%) were highest in the South. However, the West had significantly (*p* *<* 0.001) more HFpEF(+Stim) (28.77%) than HFpEF(-Stim) (15.93%). Similarly, trend analysis indicated HFpEF(+Stim) in all regions increased significantly (*p* *<* 0.001), with the West and Midwest showing the largest proportionate increase (589% and 480%, respectively) from 2008 to 2020.

**Figure 1:**
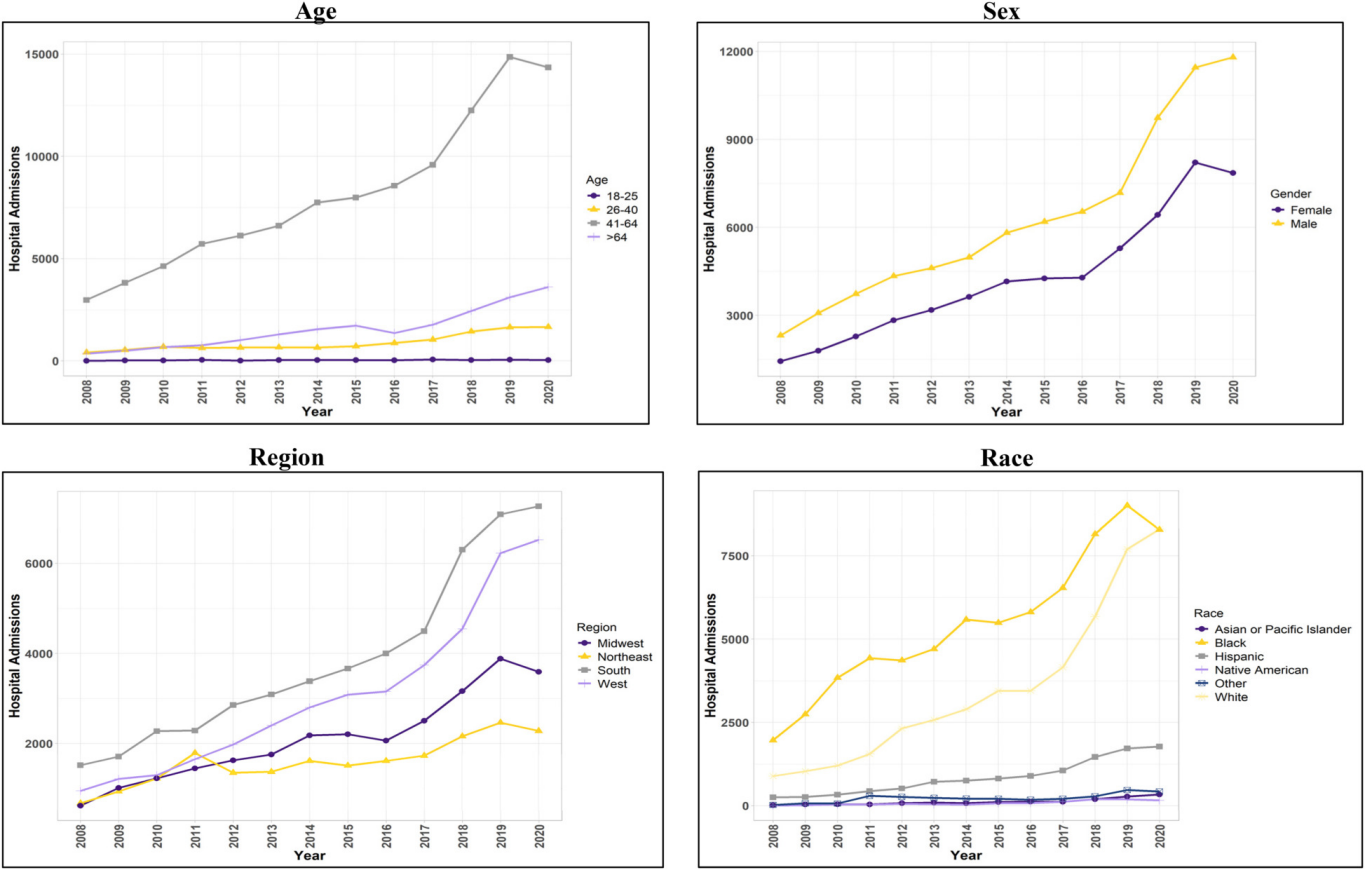
Trend in hospital admissions for patients with concurrent stimulant Use and heart failure with preserved ejection fraction.

Traditional risk factors for HF showed differences between HFpEF(+Stim) and HFpEF(-Stim). Alcohol use was significantly (*p* *<* 0.001) higher in HFpEF(+Stim) (19.68%). Hypertension was marginally higher in HFpEF(+Stim) (71.55%, *p* = 0.03) compared to HFpEF(-Stim) (71.27%). All other risk factors were significantly (*p* *<* 0.001) lower in HFpEF(+Stim) compared to HFpEF(-Stim). The results for the demographics and risk factors are available in Table 1, and the trend analysis is available in Figure 1 and Supplementary Figure 1.

**Table 1:** Characteristics in heart failure with preserved ejection fraction hospitalizations with and without stimulant-use.

Characteristics	Group	Not stimulant users Weighted *N* (%)	Stimulant users Weighted *N* (%)	*p*-value
		*N* = 18,753,926	*N* = 137,503	
Gender	Male	7,352,243 (39.21)	81,802 (59.5)	<0.001
	Female	11,400,204 (60.79)	55,680 (40.5)	
Age (in years)	18–25	17,389 (0.09)	472 (0.34)	<0.001
	26–40	225,949 (1.2)	11,620 (8.45)	
	41–64	3,850,743 (20.53)	105,218 9 (76.52)	
	>64	14,659,845 (78.17)	20,192 (14.68)	
Race/ethnicity	White	13,082,477 (73.36)	45,205 (34.07)	<0.001
	Black	2,764,861 (15.5)	70,930 (53.46)	
	Hispanic	1,182,883 (6.63)	11,011 (8.3)	
	Asian or Pacific Islander	342,727 (1.92)	1,532 (1.16)	
	Native American	86,081 (0.48)	1,036 (0.78)	
	Other	375,268 (2.1)	2,951 (2.22)	
Insurance	Medicare	15,118,606 (80.71)	48,744 (35.49)	<0.001
	Medicaid	1,213,925 (6.48)	61,358 (44.68)	
	Private Insurance	1,843,150 (9.84)	11,278 (8.21)	
	Self-Pay	268,020 (1.43)	10,693 (7.79)	
	No Charge	22,759 (0.12)	1,041 (0.76)	
	Other	266,112 (1.42)	4,213 (3.07)	
Household income (in percentile)	0–25	5,512,679 (29.86)	68,726 (52.71)	<0.001
	26–50	4,898,936 (26.54)	29,714 (22.79)	
	51–75	4,416,243 (23.92)	20,953 (16.07)	
	76–100	3,631,772 (19.67)	10,990 (8.43)	
Region	Northeast	3,969,478 (21.17)	20,715 (15.07)	<0.001
	Midwest	4,802,047 (25.61)	27,279 (19.84)	
	South	6,995,768 (37.3)	49,944 (36.32)	
	West	2,986,633 (15.93)	39,564 (28.77)	
Mortality status	Alive	17,957,749 (95.8)	134,493 (97.88)	<0.001
	Dead	786,876 (4.2)	2,916 (2.12)	
Length of stay (in days)	0–3	6,761,430 (36.05)	61,083 (44.43)	<0.001
	4–6	5,858,580 (31.24)	37,594 (27.34)	
	7–9	2,836,269 (15.12)	16,751 (12.18)	
	10–12	1,330,842 (7.10)	7,853 (5.71)	
	>12	1,966,107 (10.48)	14,215 (10.34)	
Ischemic heart disease	Present	8,239,231 (43.39)	45,570 (33.14)	<0.001
Alcohol use	Present	418,112 (2.37)	25,457 (19.68)	<0.001
Diabetes	Present	7,912,383 (44.80)	48,776 (37.71)	<0.001
Hypertension	Present	12,586,837 (71.27)	92,536 (71.55)	0.03
Obesity	Present	4,357,043 (24.67)	34,959 (27.03)	<0.001
Renal failure	Present	5,330,241 (34.24)	36,236 (33.04)	<0.001

### Demographic, risk factors, and trend analysis results for HFrEF hospital admissions

The findings described in this section all met the criterion for statistical significance (*p* *<* 0.001). HFrEF hospitalizations among stimulant users [HFrEF**(+**Stim)] increased by over 540% from 2008 to 2020. HFrEF(+Stim) and HFrEF hospitalizations among stimulant non-users [HFrEF(-Stim)] were more likely in male patients than in female patients, comprising 74.15% and 61.55%, respectively. An increase in HFrEF(+Stim) from 2008 to 2020 was observed for males (574%) and females (469%), with a more pronounced increase in males.

HFrEF(-Stim) was most prominent in patients over 64 years (69.69%). However, the majority of HFrEF(+Stim) occurred in those between the ages of 41 and 64 (72.65%). Similarly, trend analysis showed that those aged 41–64 comprised the majority of HFrEF(+Stim), though increases were seen across every age group for HFrEF(+Stim) from 2008 to 2020.

HFrEF(-Stim) was mainly comprised of White patients (69.06%), whereas HFrEF(+Stim) was mostly Black patients (43.28%) closely followed by White patients (39.58%). The trend analysis similarly showed HFrEF(+Stim) increasing from 2008 to 2020 for all race/ethnicity groups, with White and Black patients comprising the majority groups. Note that White patients surpassed Black patients in number of hospitalizations from 2018 on.

Most HFrEF(-Stim) were covered by Medicare (72.28%), while a plurality of HFrEF(+Stim) used Medicaid (47.42%) as their primary payer. Regarding household income, 59% of HFrEF(-Stim) were in the lower half of household income, while in HFrEF(+Stim), 47.42% were in the poorest (25th) percentile. The South comprised the largest proportion (40.08%) of HFrEF(-Stim). Among stimulant users, the West (36.97%) and South (32.62%) were the most common regions for patients with HFrEF. An increase in hospitalizations was seen in all regions of the United States, with the largest at 1,267% in the West. Notably, the South showed the highest HFrEF(+Stim) from 2008 to 2012 until surpassed by the West in 2013.

**Figure 2:**
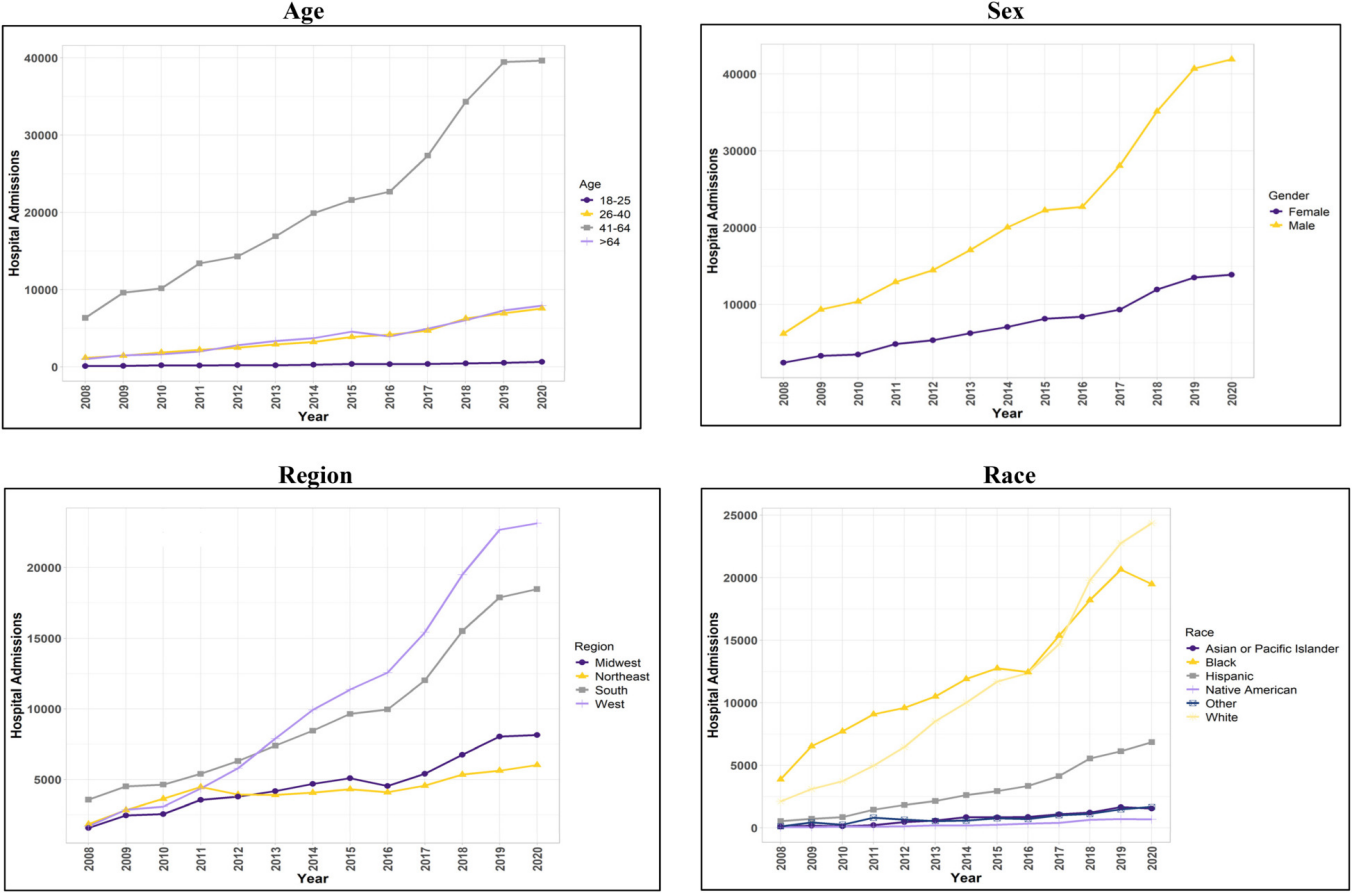
Trend in hospital admissions for patients with concurrent stimulant Use and heart failure with reduced ejection fraction.

Traditional risk factors for HF showed differences between HFrEF(+Stim) and HFrEF(-Stim). Alcohol use was higher in HFrEF(+Stim) (20.93%). Hypertension was significantly lower in HFrEF(+Stim) (59.67%) compared to HFrEF(-Stim) (65.84%). All other risk factors were significantly lower in HFrEF(+Stim) compared to HFrEF(-Stim). The results for other demographics and risk factors are shown in Table 2, and for trend analysis see Figure 2 and Supplementary Figure 2.

**Table 2:** Characteristics in heart failure with reduced ejection fraction hospitalizations with and without stimulant-use.

Characteristics	Group	Not stimulant users Weighted *N* (%)	Stimulant users Weighted *N* (%)	*p*-value
		*N* = 14,818,595	*N* = 379,349	
Gender	Male	9,120,355 (61.55)	281,249 (74.15)	<0.001
	Female	5,697,210 (38.45)	98,054 (25.85)	
Age (in years)	18–25	50,354 (0.34)	4,116 (1.08)	<0.001
	26–40	388,945 (2.62)	48,838 (12.87)	
	41–64	4,200,326 (28.34)	275,596 (72.65)	
	>64	10,178,970 (68.69)	50,799 (13.39)	
Race/ethnicity	White	9,669,413 (69.06)	144,556 (39.58)	<0.001
	Black	2,590,976 (18.51)	158,075 (43.28)	
	Hispanic	1,040,469 (7.43)	39,057 (10.69)	
	Asian or Pacific Islander	259,703 (1.85)	9,688 (2.65)	
	Native American	81,169 (0.58)	3,790 (1.04)	
	Other	359,070 (2.56)	10,084 (2.76)	
Insurance	Medicare	10,695,765 (72.28)	105,135 (27.77)	<0.001
	Medicaid	1,370,463 (9.26)	179,521 (47.42)	
	Private Insurance	1,990,030 (13.45)	39,546 (10.45)	
	Self-Pay	411,245 (2.78)	38,639 (10.21)	
	No Charge	34,458 (0.23)	2,779 (0.73)	
	Other	295,604 (2)	12,984 (3.43)	
Household income (in percentile)	0–25	4,829,472 (33.25)	168,322 (47.38)	<0.001
	26–50	3,884,898 (26.75)	83,406 (23.48)	
	51–75	3,300,533 (22.73)	63,567 (17.89)	
	76–100	2,508,798 (17.27)	39,988 (11.26)	
Region	Northeast	3,008,186 (20.3)	54,618 (14.4)	<0.001
	Midwest	3,490,203 (23.55)	60,733 (16.01)	
	South	5,939,370 (40.08)	123,747 (32.62)	
	West	2,380,836 (16.07)	140,250 (36.97)	
Mortality status	Alive	14,055,811 (94.9)	368,657 (97.24)	<0.001
	Dead	756,110 (5.1)	10,459 (2.76)	
Length of stay (in days)	0–3	5,637,243 (38.04)	172,439 (45.46)	<0.001
	4–6	4,414,400 (29.80)	107,750 (28.40)	
	7–9	2,134,582 (14.40)	44,344 (11.70)	
	10–12	1,028,517 (6.94)	19,898 (5.24)	
	>12	1,603,414 (10.82)	34,909 (9.20)	
Ischemic heart disease	Present	9,190,111 (62.02)	152,961 (40.32)	<0.001
Alcohol use	Present	521,012 (3.75)	74,230 (20.93)	<0.001
Diabetes	Present	5,909,810 (42.54)	101,878 (28.73)	<0.001
Hypertension	Present	9,146,253 (65.84)	210,544 (59.37)	<0.001
Obesity	Present	2,193,650 (15.79)	53,174 (14.99)	<0.001
Renal failure	Present	4,141,041 (33.2)	72,612 (24.3)	<0.001

### Adjusted odds ratio for HFpEF hospital admissions

The first model was used to determine the likelihood of being hospitalized with HFpEF associated with stimulant use. It showed that stimulant use was less likely (OR = 0.96, CI 0.94–0.98) to be associated with HFpEF after adjusting for traditional risk factors for HF and demographics.

**Figure 3:**
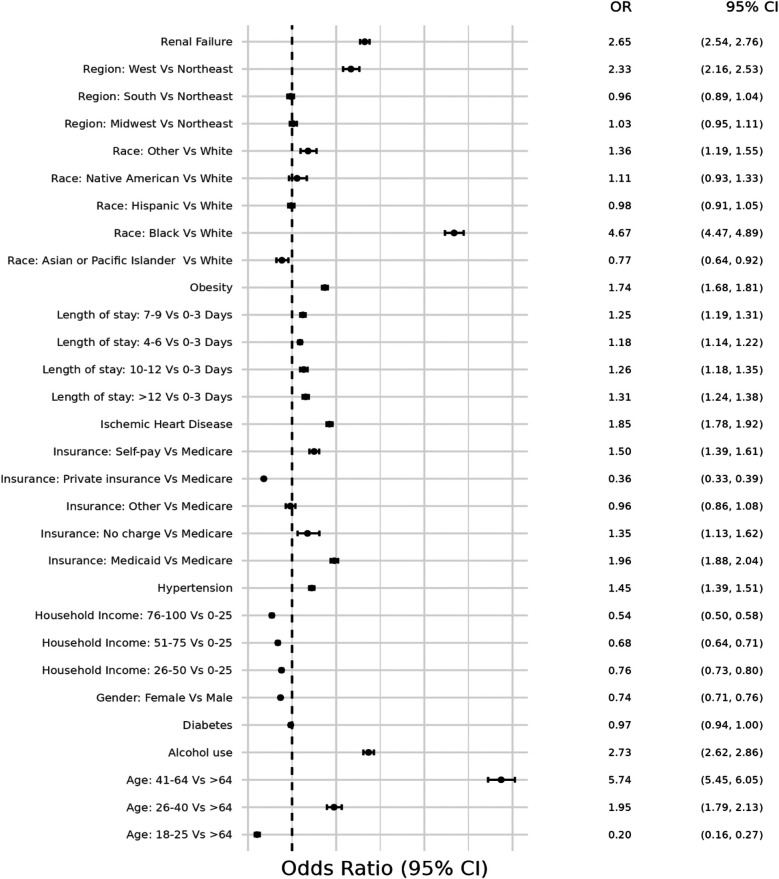
Odds ratio plot for demographics and risk factors in hospital admissions with concurrent stimulant Use and heart failure with preserved ejection fraction.

The second model determined the at-risk demographic groups and traditional risk factors for HF that were more likely to be associated with HFpEF(+Stim) patients. Patients ages 41–64 years had the highest likelihood (OR = 5.74, CI 5.45–6.05), and female patients had a lower likelihood (OR = 0.74, CI 0.71–0.76) of hospitalization than male patients. Black patients were more likely (OR = 4.67, CI 4.47–4.89) to be hospitalized than White patients, whereas non-Hispanic Asian (hereafter Asian) patients were less likely (OR = 0.77, CI 0.64–0.92) to be hospitalized than White patients. Hispanic and non-Hispanic Native American/Alaskan Native (hereafter Native American) patients had similar odds (OR = 0.98, CI 0.91–1.05; OR = 1.11, CI 0.93–1.33, respectively) compared to White patients. HFpEF(+Stim) were more likely to be covered by Medicaid (OR = 1.96, CI 1.88–2.04) or self-pay (OR = 1.50, CI 1.39–1.61) for their hospitalizations. Compared to the Northeast, the West had higher odds (OR = 2.33, CI 2.16–2.53) of hospitalizations, while other regions had similar odds to the Northeast. Patients in the 0–25th percentile household income were more likely to be hospitalized, with odds decreasing as household income increases, with the lowest likelihood in patients in the >75th percentile (OR = 0.54, CI 0.50–0.58). HFpEF(+Stim) patients were more likely to suffer from risk factors, except diabetes (OR = 0.97, CI 0.94–1.00). The odds ratio plot for HFpEF(+Stim) is shown in Figure 3.

### Adjusted odds ratio for HFrEF hospital admissions

Similarly to HFpEF, the first model was used to determine the likelihood of being hospitalized with HFrEF due to stimulant use. It demonstrated that stimulant users were more likely (OR = 1.97, CI 1.93–2.01) to be hospitalized with HFrEF compared to non-users, after adjusting for traditional risk factors for HF and demographic characteristics.

**Figure 4:**
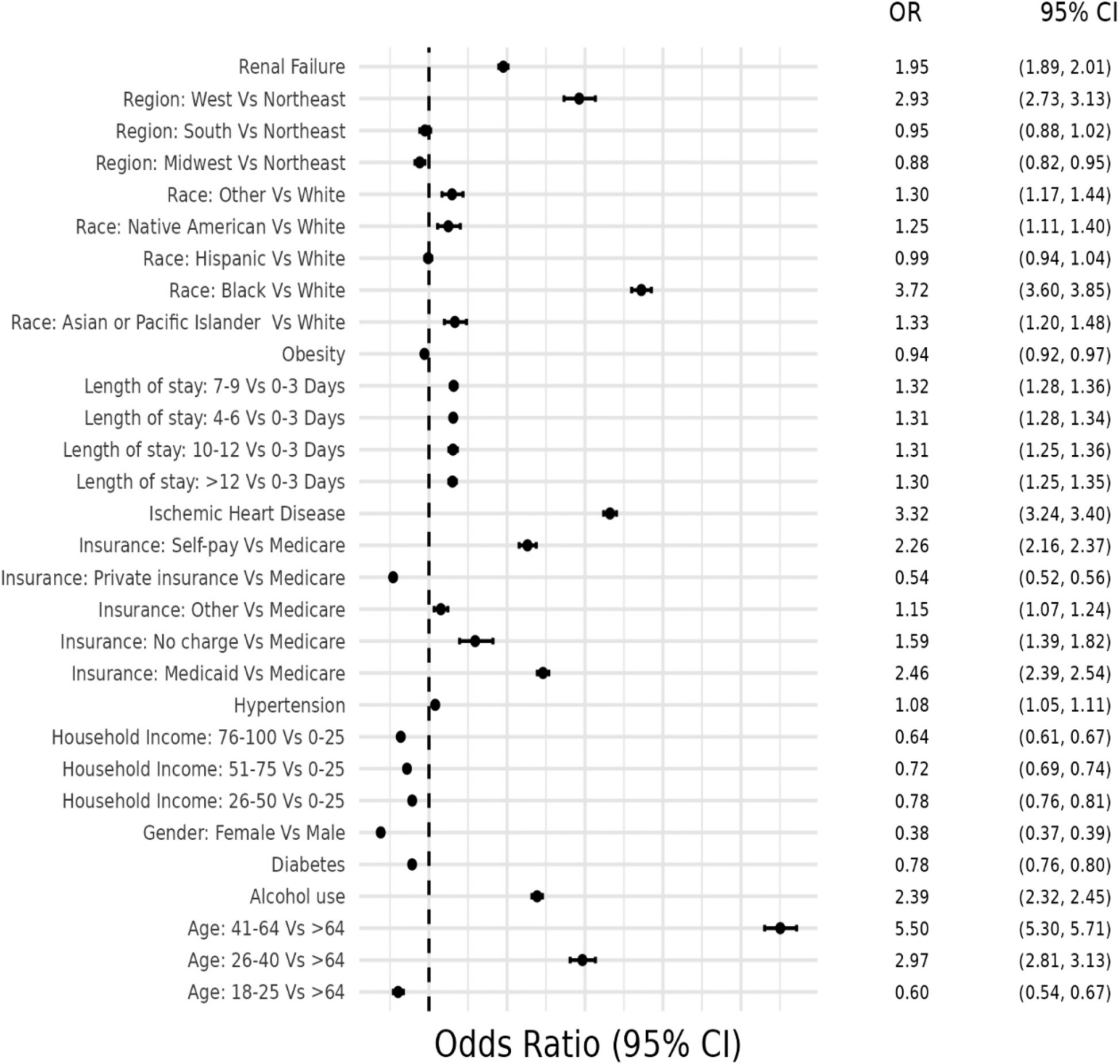
Odds ratio plot for demographics and risk factors in hospital admissions with concurrent stimulant Use and heart failure with reduced ejection fraction.

The second model examined at-risk demographic groups and associated traditional risk factors for HF that were more likely to be associated with HFrEF(+Stim) patients. Patients aged 41–64 years had the highest likelihood (OR = 5.50, CI 5.30–5.71), and female patients had a lower likelihood of hospitalization than male patients (OR = 0.38, CI 0.37–0.39). Except for Hispanic patients, every other racial/ethnic group had a higher likelihood of hospitalization compared to White patients, with the highest odds in Black patients (OR = 3.72, 3.60–3.85). HFrEF(+Stim) were more likely to be covered by Medicaid (OR = 2.46, CI 2.39–2.54) or self-pay (OR = 2.26, CI 2.16–2.37). Compared to the Northeast, the West had higher odds (OR = 2.93, CI 2.73–3.13) of HFrEF(+Stim), while other regions had similar odds to the Northeast. Patients in the 0–25th percentile household income were more likely to be hospitalized, with odds decreasing as household income increases, with the lowest likelihood in patients in the >75th percentile (OR = 0.64, CI 0.61–0.67). HFrEF(+Stim) patients were more likely to suffer from risk factors, except diabetes (OR = 0.97, CI 0.94–1.00) and obesity (OR = 0.94, CI 0.92–0.97). The odds ratio plot for HFrEF(+Stim) is shown in Figure 4.

## Discussion

The systematic analysis of NIS data in the present study has advanced our understanding of ejection fraction in heart failure for individuals admitted to the hospital with or without current stimulant use. For such individuals, we identified demographic patterns associated with types of HF as well as a more complete picture of the their associations as seen from 2008 to 2020. There was a profound increase among all types of stimulant-related HF hospitalizations—423% vs. 540% for preserved and reduced ejection fraction, respectively. This increase was seen in all age groups and both sexes but varied by identified race/ethnicity. Though the increase was mostly uniform, there was a slight deceleration from 2019 to 2020, perhaps reflecting oversaturation with COVID hospitalizations. Overall, this summary of the outcomes illustrates the severity of the stimulant use epidemic in the US and the need to better understand its connection between stimulant-related HF and ejection fraction (34).

The generalized linear model adjusted for stimulant use, demographics, and traditional risk factors for HF indicated that stimulant use was associated with an increased likelihood of hospitalizations from HFrEF but a lower likelihood with HFpEF. The increased odds ratio for HFrEF is consistent with other reports of HFrEF in patients using stimulants (19). Similarly, there have been cases of HFpEF reported among stimulant users (19). Here, we found a lower likelihood of HFpEF among stimulant users compared to non-stimulant users. Based on these findings, we suggest that stimulant-associated HFpEF patients are likely to progress to HFrEF with continued stimulant use. Both amphetamines and cocaine act as sympathomimetics, increasing heart rate, blood pressure, and free radicals. Amphetamines have a longer duration of action and show direct cardiotoxicity, which is exacerbated by concurrent stimulation of catecholamine production (35). Furthermore, cessation of stimulant use has led to improvement in ejection fraction among HFrEF(+Stim) patients (36, 37). Future research investigating the nature of the damage leading to persistent myocardial dysfunction in stimulant users will help design approaches aimed at improving treatment outcomes.

Examination of age found that in stimulant users, HF, irrespective of type, developed among the middle-aged (those aged 41–64 years), followed by individuals over 64 years, while in non-users HF developed later in life (>64 years), followed by middle age (41–64 years). This outcome supports the suggestion that stimulant use accelerates heart tissue damage (7, 38), leading to HF at an earlier age. One question of interest is whether long-term cessation from stimulant use could allow at least some reversal of such heart tissue damage and, if so, what intervention regimens would facilitate this reversal.

Additionally, from 2008 to 2020 there was a significant increase in stimulant-related HF of all types across the four age groups included in this study. This observation prompts several points of discussion. First, the increase in patients aged 18–25 (about 5–7 times) indicates that younger population appear to be increasing their use of stimulants and/or using in a more dangerous manner, as this age group comprises a significantly smaller portion of HF cases without stimulant use. While future work will need to systematically parse out these different possibilities, this outcome supports the earlier suggestion that stimulant use accelerates HF. Second, the HF pattern across age groups points to a possible cohort effect due to the chronic use of stimulants at a much younger age (<41 years) leading to the development of heart failure in middle age (41–64 years) and old age (>64 years) (25, 26, 39). These combined effects lead to younger patients with higher disease burden in the long-term, which underlines the severity of stimulant-related HF.

Analysis of sex as reported in the NIS revealed that stimulant-related and non-stimulant-related HF hospitalizations were more prevalent in male patients for all HF groups, except for HFpEF(-Stim) patients. The female predominance of HFpEF has been noted previously and hypothesized to be caused by increased inflammatory response and microvascular and endothelial dysfunction (40–42). In contrast, among stimulant users, male patients had a higher overall prevalence, especially in HFrEF, across our analyses. This prevalence of HFrEF in males has been noted previously and hypothesized to be caused by macrovascular coronary artery disease, myocardial infarction, and higher rates of apoptosis, necrosis, and cardiomyocyte loss in myocardial infarction (41, 42). We propose that the higher male prevalence in stimulant-related HFrEF reflects an acceleration of these processes driven by higher stimulant use among males, similar to our findings by age distribution (43). The finding that HFpEF(+Stim) is also higher in males is an interesting observation. We suspect that this could be due to the mechanism of stimulant-induced cardiomyopathy, which is not well understood but may involve proinflammatory responses and cardiac remodeling (7).

Examination of race/ethnicity as reported in the NIS found for stimulant-induced HF hospitalizations that Black patients had the highest prevalence in every HF category, followed by White and Hispanic patients. In our trend analysis, it is notable that Hispanic and White patients are becoming more vulnerable to stimulant-related HF. In fact, White patients categorized as HFrEF(+Stim) exceeded Black patients in 2018, and the same occurred in HFpEF(+Stim) by 2020. This pattern replicates an earlier report with socioeconomic status being implicated in Black HF hospitalizations (22, 25, 44). Additionally, stigma plays an important role in the racial differences seen in methamphetamine use disorder and mental health (21). These patients likely experience a synergistic effect of stress from experiences of racial/ethnic discrimination along with that of an individual with stimulant use disorder (45). In contrast, the increasing prevalence in White and Hispanic patients is thought to be driven by increased stimulant use (23, 25, 43). These racial differences highlight the importance of targeted substance use treatments and further investigation on possible socio-cultural and biological mechanisms for racial/ethnic disparities in stimulant-associated HF (46, 47).

Examining the primary payers for HF hospitalizations showed that Medicare covered most non-users. This is unsurprising, given that most HF patients in the non-user groups were at least 65 years old. However, among stimulant users, Medicaid-insured patients comprised the majority in every analysis. This outcome might reflect that most stimulant-using HF patients were in the lower half of household income. Stimulant users were also more likely to self-pay for their medical costs than non-users. This finding may also be related to household income. Non-users’ income had a relatively uniform distribution across income brackets. In contrast, stimulant users mostly belonged to the two lowest two quartiles– 0–25th and 26–50th percentiles. This distribution suggests that stimulant-associated HF patients are more likely to be economically disadvantaged, which again demonstrates the synergistic effect of HF and stimulant use disorder, as both are conditions associated with low socioeconomic status (40, 43, 44, 48–50).

The geographic distribution of hospitalized HF patients was notable. The highest prevalence of HF patients among non-users was in the South. In stimulant users, HFpEF was most prevalent in the South, followed by the West. However, this prevalence was flipped for HFrEF which was higher in the West, followed by the South. Though all regions showed significant increases from 2008 to 2020, the West had the largest proportionate increase of stimulant-related HF of all types, exceeding Southern stimulant-related HFrEF hospitalizations by 2013. In the South, these hospitalizations are likely due to a synergistic effect of already poor cardiovascular health with increasing stimulant use (47, 51). In the West, this is likely due to a traditionally high prevalence of stimulant use that continues to increase (47). Future research would benefit from a more detailed understanding within each region of the association between ejection fraction in HF and stimulant use.

The influence of other HF risk factors in stimulant-related HF was also investigated. Alcohol use showed increased prevalence in the bivariate analysis with the models showing higher odds of use in both HFrEF(+Stim) and HFpEF(+Stim) patients. Polysubstance use among stimulant users is well-known, and alcohol use can also cause HF through cardiotoxicity (35, 52, 53). In our study, we suggest that stimulant use has independently increased the risk of HFrEF. This suggestion is based on the finding that alcohol use was significantly higher in both HFrEF(+Stim) and HFpEF(+Stim) patients. Other traditional risk factors for HF show an interesting difference between bivariate and model analysis among stimulant-associated HF hospitalizations. Risk factors, such as diabetes, hypertension, ischemic heart disease, and renal failure were lower among stimulant-associated HF of all subtypes in the bivariate analysis which is in alignment with that observed in other studies (26, 48), but in the model, they had a higher likelihood of being associated with HFpEF or HFrEF with concurrent stimulant use. We hypothesize that even though these risk factors are less prevalent among stimulant users, possibly due to the higher prevalence of HF among the younger population in stimulant users, continued stimulant use may increase the risk of hospitalization with HF, complicating and exacerbating HF.

This study offers valuable insights into demographic patterns for stimulant-induced HF and their subtypes along with associated risk factors. These findings provide a foundation for future longitudinal research aimed at establishing causal relationships between stimulant use and HF, as well as identifying additional, previously unrecognized risk factors. Such research could inform the development of more effective treatment guidelines and targeted preventive strategies to mitigate the onset and progression of HF in stimulant users. Furthermore, these measures can strengthen current public health initiatives focused on rehabilitation and recovery of stimulant users, increasing vigilance for the risk for cardiovascular diseases, especially HF. Ultimately, this could help reduce healthcare costs associated with treatment for complications of continued stimulant use, particularly when many users lack adequate healthcare coverage and belong to lower socioeconomic groups.

## Conclusion

Stimulant-associated HF hospitalizations are increasing across all subtypes, with a positive association between stimulant use and HFrEF. We found that stimulant-associated HF accelerates HF, with stimulant-associated HF of all types occurring at younger ages than non-stimulant-associated HF. This outcome may reflect different pathophysiological mechanisms from traditional HF, as HFpEF is typically not seen in males, but HFpEF(+Stim) showed higher prevalence in males. In addition, stimulant-related HF of all subtypes had higher odds of suffering from traditional risk factors of HF, adding to the risk. In many of these patients, stimulant use and HF likely play a combinatoric effect on outcomes, especially among the socioeconomically disadvantaged. Furthermore, the changing trends among racial and regional differences in stimulant-associated HF highlight the need for further research, targeted treatments, and additional resources to combat the increasing burden of stimulant-associated HF.

## Data Availability

The dataset used in this study is derived from the National Inpatient Sample (NIS), which is a proprietary dataset available through the Healthcare Cost and Utilization Project (HCUP). Due to data use agreements and licensing restrictions, we are not permitted to publicly share the raw data. Requests to access these datasets can be obtained here: https://hcup us.ahrq.gov/db/nation/nis/nisdbdocumentation.jsp.
