# Supplementary material for: Hospitalized patients and stimulant use-associated heart failure: importance of ejection fraction and related risk factors
Source: Front Cardiovasc Med. 2025 Jul 3;12:1566481. doi: 10.3389/fcvm.2025.1566481 (PMC12267280; doi:10.3389/fcvm.2025.1566481)
Supplement: Supplementary file 1 [file Datasheet1.docx]

**Hospitalized Patients and Stimulant Use-Associated Heart Failure: Import of Ejection Fraction and Related Risk Factors**

Akshat Agrawal, MBBS^1#^, Brooke Scardino, B.S.^2#^, Diensn G Xing, BSc^2#^, Md. Shenuarin Bhuiyan, PhD^3,4^, Rick A. Bevins, Ph.D^5^, Kalgi Modi, MD^2^, Tarek Helmy, MD^2^, Steven A. Conrad, MD, PhD^2,8^, Nicholas E. Goeders, PhD^6^, Md Mostafizur Rahman Bhuiyan, MD^7^, John A. Vanchiere, MD, PhD^8^, A Wayne Orr, PhD^3,4^, Christopher G. Kevil, PhD ^3,4^, Mohammad Alfrad Nobel Bhuiyan, PhD ^2,4,9^*

^1^Department of Public Health, Louisiana State University Health Sciences Center at Shreveport. Shreveport, LA, 71103, USA

^2^Department of Medicine, Louisiana State University Health Sciences Center at Shreveport, Shreveport, LA, 71103, USA

^3^Department of Molecular and Cellular Physiology, Louisiana State University Health Sciences Center at Shreveport, Shreveport, LA, 71103, USA

^4^Department of Pathology and Translational Pathobiology, Louisiana State University Health Sciences Center at Shreveport, Shreveport, LA, 71103, USA

^5^Department of Psychology, University of Nebraska-Lincoln, Lincoln, Nebraska, 68588-0308, USA

^6^Department of Pharmacology, Toxicology & Neuroscience, Louisiana State University Health Sciences Center at Shreveport, Shreveport, LA, 71103, USA

^7^Department of Pediatric Cardiology, Bangabandhu Sheikh Mujib Medical University, Dhaka, Bangladesh

^8^Department of Pediatrics, LSU Health Sciences Center Shreveport, Shreveport, LA, 71103, USA

^9^Louisiana Addiction Research Center, Louisiana State University Health Sciences Center at Shreveport, Shreveport, LA, USA

# These authors contributed equally.

***Correspondence to:** Mohammad Alfrad Nobel Bhuiyan, PhD, Division of Clinical Informatics, Department of Medicine, Louisiana State University Health Sciences Center, PO Box 33932, Shreveport, LA 71130-3932. Email: [Nobel.Bhuiyan@lsuhs.edu](mailto:Nobel.Bhuiyan@lsuhs.edu)

**Supplementary Tables**

**Supplementary Table S1: ICD-9 and ICD-10 codes for Heart Failure**

| **Diagnosis** | **ICD-9** | **ICD-10** |
| --- | --- | --- |
| Heart failure with reduced ejection fraction [HFrEF]  (unspecified) | 428.2 | I50.20 |
| Heart failure with reduced ejection fraction [HFrEF]  (acute) | 428.21 | I50.21 |
| Heart failure with reduced ejection fraction [HFrEF]  (chronic) | 428.22 | I50.22 |
| Heart failure with reduced ejection fraction [HFrEF]  (acute on chronic) | 428.23 | I50.23 |
| Heart failure with preserved ejection fraction [HFpEF]  (unspecified) | 428.3 | I50.30 |
| Heart failure with preserved ejection fraction [HFpEF]  (acute) | 428.31 | I50.31 |
| Heart failure with preserved ejection fraction [HFpEF]  (chronic) | 428.32 | I50.32 |
| Heart failure with preserved ejection fraction  [HFpEF]  (acute on chronic) | 428.33 | I50.33 |

**Supplementary Table S2: ICD-9 and ICD-10 codes for Methamphetamine and Cocaine Use**

| **Diagnosis** | **ICD-9** | **ICD-10** |
| --- | --- | --- |
| Methamphetamine mild use disorder (abuse) | 305.70 or 305.71 or 305.72 | F15.10 |
| Methamphetamine moderate or severe use disorder (dependence) | 304.40 or 304.41 or 304.42 | F15.20 |
| Methamphetamine mild use disorder (abuse), in remission | 305.73 | F15.11 |
| Methamphetamine moderate or severe use disorder (dependence), in remission | 304.43 | F15.21 |
| Poisoning by amphetamines, accidental (unintentional), initial encounter | 969.72 | T43.621A |
| Poisoning by amphetamines, intentional self-harm, initial encounter | 969.72 | T43.622A |
| Poisoning by amphetamines, assault, initial encounter | 969.72 | T43.623A |
| Poisoning by amphetamines, undetermined, initial encounter | 969.72 | T43.624A |
| Poisoning by methamphetamines intentional self-harm, initial encounter | NA | T43.652A |
| Poisoning by methamphetamines intentional self-harm, subsequent encounter | NA | T43.652D |
| Poisoning by methamphetamines intentional self-harm, sequela | NA | T43.652S |
| Adverse effect of methamphetamines, subsequent encounter | NA | T43.655D |
| Adverse effect of methamphetamines, sequela | NA | T43.655S |
| Cocaine mild use disorder (abuse) | 305.60 or 305.61 or 305.62 | F14.10 |
| Cocaine mild abuse disorder(abuse), in remission | 305.63 | F14.11 |
| Cocaine moderate or severe use disorder (dependence) | 304.20 or 304.21 or 304.22 | F14.20 |
| Cocaine moderate or severe use disorder (dependence), in remission | 304.23 | F14.21 |
| Poisoning by cocaine, intentional self-harm, initial encounter | 970.81 | T40.5X2A |
| Poisoning by cocaine, assault, initial encounter | 970.81 | T40.5X3A |
| Poisoning by cocaine, undetermined, initial encounter | 970.81 | T40.5X4A |

**Supplementary Table S3: Names of the states in each region (the southern region includes the District of Columbia, the seat of the federal government of the United States).**

| **Region** | **States** |
| --- | --- |
| Northeast | Connecticut, Maine, Massachusetts, New Hampshire, New Jersey, New York, Pennsylvania, Rhode Island, Vermont |
| Midwest | Illinois, Indiana, Iowa, Kansas, Michigan, Minnesota, Missouri, Nebraska, North Dakota, Ohio, South Dakota, Wisconsin |
| South | Alabama, Arkansas, Delaware, Florida, Georgia, Kentucky, Louisiana, Maryland, Mississippi, North Carolina, Oklahoma, South Carolina, Tennessee, Texas, Virginia, West Virginia  Washington, DC |
| West | Alaska, Arizona, California, Colorado, Hawaii, Idaho, Montana, Nevada, New Mexico, Oregon, Utah, Washington, Wyoming |

**Supplementary Figure 1: Trend by Number of Cases in Hospital Admissions with Concurrent Stimulant Use and Heart Failure with Preserved Ejection Fraction**

**
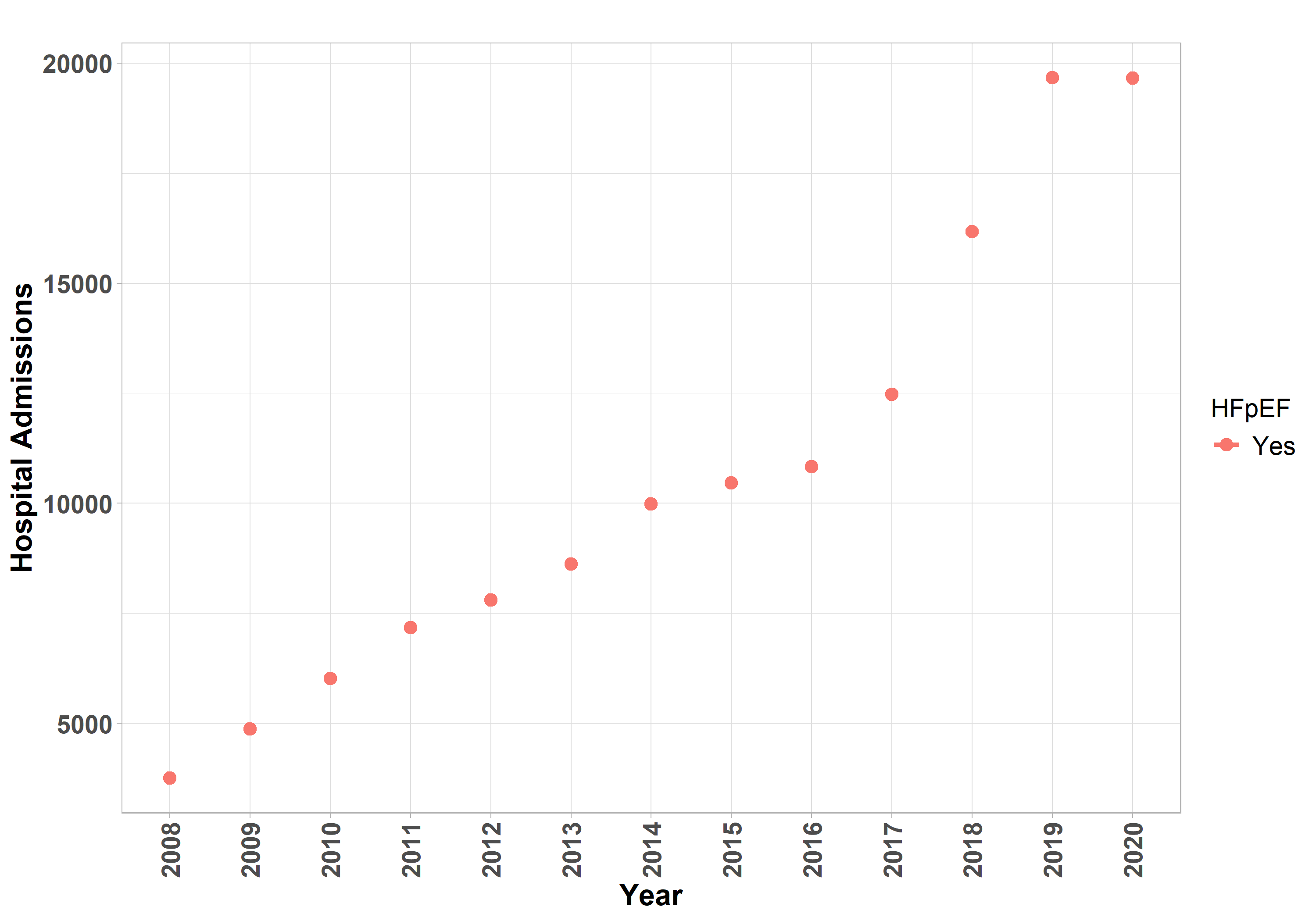
**

**Supplementary Figure 2: Trend by Number of Cases in Hospital Admissions with Concurrent Stimulant Use and Heart Failure with Reduced Ejection Fraction
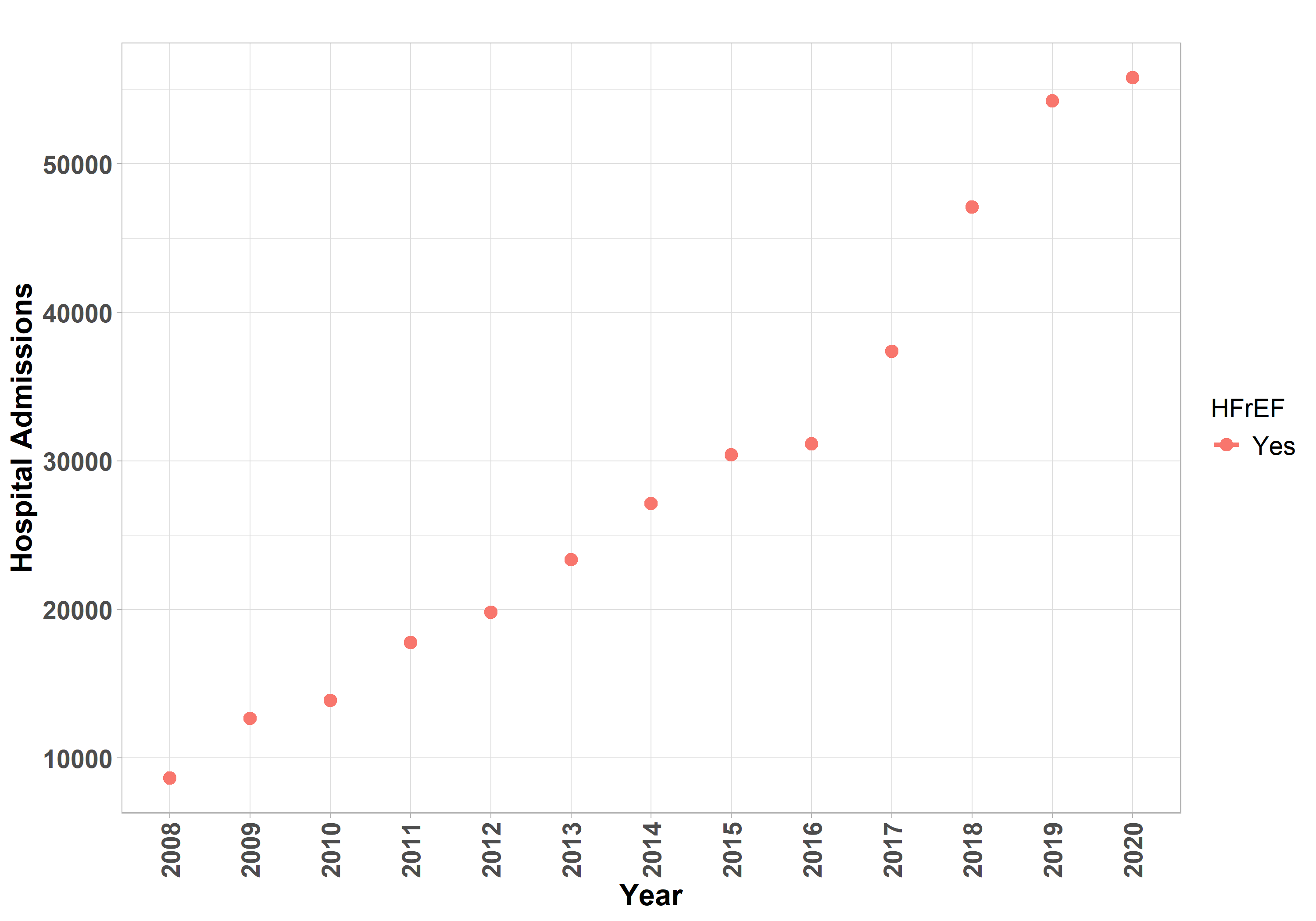
**
